# Is Glucose-6-Phosphate Dehydrogenase Deficiency a Risk Factor for Autoimmune Thyroid Disease? A Retrospective Case–Control Study

**DOI:** 10.3390/ijerph20032709

**Published:** 2023-02-03

**Authors:** Maria Pina Dore, Giuseppe Fanciulli, Giovanni Mario Pes

**Affiliations:** 1 Dipartimento di Medicina, Chirurgia e Farmacia, Clinica Medica, University of Sassari, Viale San Pietro 8, 07100 Sassari, Italy; 2 Baylor College of Medicine, One Baylor Plaza Blvd, Houston, TX 77030, USA; 3 Endocrine Unit, AOU Sassari, 07100 Sassari, Italy; 4 Sardinia Longevity Blue Zone Observatory, 08040 Ogliastra, Italy

**Keywords:** autoimmune thyroid disease, glucose-6-phosphate dehydrogenase deficiency, oxidative stress

## Abstract

Background: The risk of developing thyroid disorders (TDs) in subjects with inherited glucose-6-phosphate dehydrogenase (G6PD) deficiency is unknown. The aim of this study was to explore the association between autoimmune (AITD) and G6PD deficiency in Northern Sardinia, in a population with a high frequency of these two conditions. Methods: In this retrospective single-center case–control study, demographic and clinical data were collected from patients examined in a tertiary referral Gastroenterology Section of a teaching hospital. Results: In 8894 subjects examined (64.7% females), 1218 patients were diagnosed with TDs; more specifically, 767 were diagnosed with AITD and 451 were not (non-AITD). Overall, G6PD deficiency was more prevalent in TD patients compared with patients without TD (controls) (16.7% vs. 11.2%; *p* < 0.0001). Multivariable logistic regression analysis (after adjusting for age, sex, excess weight and smoking habits), confirmed a higher risk of AITD among G6PD deficient patients with an odds ratio (OR) of 1.36 and 95% confidence interval (CI) of 1.11–1.6, female patients (OR 1.33, 95% CI 1.07–1.65) and overweight patients (OR 1.22, 95% CI 1.03–1.44). Conclusions: The risk of AITD is increased in carriers of G6PD deficiency. A careful assessment of thyroid function is advisable in patients with inherited G6PD defects.

## 1. Introduction

The relationship between thyroid function and oxidative metabolism is complex and poorly understood [1]. It has long been known that, inside thyroid follicular cells, the biosynthesis of the hormones triiodothyronine (3,3′,5-triiodo-L-thyronine, T3) and thyroxine (3,5,3′,5′-tetraiodothyronine, T4) requires a series of redox reactions, which have reactive oxygen species (ROS) as byproducts [2]. These redox reactions require the availability of an adequate amount of hydrogen peroxide (H_2_O_2_) [3], itself a ROS, albeit not a free radical, as it does not contain unpaired electrons. It has been demonstrated that H_2_O_2_ is generated in the apical membrane of thyrocytes [4]. Recently, investigations regarding the precise site of ROS production in the thyroid gland led to the identification of dual oxidase enzymes (DUOX1 and 2) [5]. These enzymes, belonging to the nicotinamide adenine dinucleotide phosphate (NADPH) oxidase (NOX) family, require NADPH as an electron donor [6]. The continuous generation of H_2_O_2_, essential for hormone biosynthesis, makes thyrocytes extremely vulnerable to oxidative stress, requiring a delicate balance between ROS overproduction and anti-oxidant defense [7]. When the amount of ROS produced exceeds the scavenging capacity of follicle antioxidant systems, cells may be exposed to the ROS’ harmful effects. Interestingly, studies performed in animal models [8] and humans [9,10] have implicated an excessive production of ROS in the onset of certain autoimmune thyroid diseases (AITDs).

Among the major metabolic pathways enabling thyroid follicles to counteract oxidative stress, there are enzymes ensuring a high rate of intracellular reduced glutathione (GSH). Evidence from experimental studies in rodents suggests that glutathione depletion may lead to thyroid dysfunction [11]. This condition may be exacerbated by a concomitant dietary selenium deficiency, which inhibits selenoenzymes involved in this antioxidant activity and, in turn, impairs thyroid hormone metabolism [12]).

Among the enzymes providing an efficient antioxidant defense by maintaining high intracellular levels of GSH, glucose-6-phosphate dehydrogenase (G6PD) is one of the most significant [13,14]. G6PD catalyzes the rate-limiting step of the pentose phosphate pathway (PPP) providing the reducing equivalents via the action of the NADPH-dependent glutathione reductase [15], maintaining GSH in the reduced form. Inherited mutations impairing G6PD catalytic activity primarily affect the red blood cells, as they lack alternative sources of NADPH [16].

G6PD deficiency is the most common enzymopathy of red blood cells and affects 400 to 500 million individuals [14]. It occurs most often in tropical and subtropical areas such as Africa, Europe, Asia, with prevalences up to 20% or more in some regions. For example, in Sardinia, Italy, the prevalence ranged from 4 to 35%, depending on the geographic area [17].

Clinical manifestations of G6PD deficiency include a spectrum of disorders from the likelihood of developing neonatal jaundice to hemolysis. Depending on the degree of the enzyme deficiency, which in turn is determined by the specific G6PD variant, deficient individuals with the most common G6PD variants (i.e., G6PD A– and G6PD Mediterranean) are usually asymptomatic. However, exposure to some medicines, such as aspirin, foods, especially fava beans, and infections may trigger severe hemolytic crisis [13]. Therefore, each newborn undergoes G6PD-activity screening in Sardinia and in several areas of the Mediterranean basin.

Besides RBCs, other organs and systems could be affected by G6PD deficiency, including the brain [18], the liver [19] and the cardiovascular system [20].

The thyroid is an organ vulnerable to oxidative challenges. Given the crucial role of G6PD in antioxidant defence, in subjects with a hereditary form of G6PD, it is reasonable to hypothesize thyroid impairment. Despite the essential role of G6PD within the antioxidant mechanisms in most tissues, studies evaluating the impact of G6PD deficiency on thyroid function in humans are lacking.

In Sardinia, a high prevalence of the hereditary G6PD deficiency has been reported in the general population (nearly 1:8) due to the past malaria endemic. Moreover, an exceptionally high number of cases with autoimmune diseases, including AITDs, has been observed, making Sardinia the ideal scenario to test the relationship between G6PD deficiency and AITDs [21].

In this case–control study, we aimed to explore the association between thyroid disorders (TDs) and G6PD deficiency in a population from Northern Sardinia, Italy.

## 2. Materials and Methods

### 2.1. Study Design

This was a retrospective single-center case–control study aimed to evaluate the association between TDs and G6PD deficiency, controlling for potential confounders, in patients examined in a tertiary referral Gastroenterology Section of a teaching hospital (University of Sassari, Italy). Patients with defined diagnosis of TDs were considered cases, and subjects without TDs were considered controls.

### 2.2. Data Collection

Data were collected from clinical records of patients who complained of gastrointestinal symptoms between 2010 and 2019, and whose accurate medical histories were available. Patients’ evaluations encompassed information such as sex, age, smoking habits, anthropometric parameters and any diagnosed TDs (including possible medications related to TD). This database has previously been used for studies involving TD [22,23,24,25].

### 2.3. Thyroid Disorders 

Thyroid-related conditions were diagnosed by the specialist endocrinologist according to current national and international guidelines/expert consensuses progressively developed, as previously described [24]. More specifically, TDs were stratified in two subgroups, AITD and non-AITD. AITD was diagnosed upon demonstration of circulating autoantibodies against thyroid antigens (specifically thyroid peroxidase antibodies (TPO-Ab)) and antibodies directed against thyroglobulin (TG-Ab), reduced echogenicity on thyroid ultrasound and/or evidence of lymphocytic infiltration on thyroid cytology/biopsy (when necessary), regardless of the presence of clinical symptoms. Based on the laboratory and/or pathological thyroid profile, patients with AITD were further classified as having either Hashimoto’s thyroiditis or Graves’ disease. Non-AITD patients included patients with drug-induced dysthyroidism, thyroidectomy (including partial or subtotal thyroidectomy), isolated thyroid nodule and non-toxic multinodular goiter.

### 2.4. Glucose-6-Phosphate Dehydrogenase Deficiency

The condition of G6PD deficiency was recorded for each participant based on the results of previous laboratory tests in which a reduction in the glucose-6-phosphate dehydrogenase/6-phosphogluconate dehydrogenase (G6PD/6PGD) ratio in red blood cells of peripheral venous blood was detected after adjustment for hemoglobin levels, according to a previously described method [26]. The conditions of severe deficiency (<10% residual G6PD activity) and intermediate deficiency (between 10% and 90% residual activity) were collapsed into the same category. Molecular tests for the inherited G6PD deficiency were not available.

### 2.5. Statistical Analysis

Patients with TDs and without TDs were compared according to the study variables and G6PD status using the Student’s t test for scalar variables and using the Pearson χ^2^ test for categorical variables. According to their smoking habits, patients were stratified in two categories, as never smoked or former and current smokers. The body mass index (BMI) was calculated using the formula weight (kg)/height (m)^2^, and obesity was defined as a BMI ≥ 30 kg/m^2^. Patients with severe and intermediate G6PD deficiency were grouped together as G6PD deficient. Moreover, the association between AITD and G6PD deficiency was determined using univariable and multivariable logistic regression models.

Additional analyses were conducted regarding AITD and non-AITD study participants. Unadjusted and adjusted odds ratio (ORs) and their 95% confidence intervals (CI) were calculated. All statistical analyses were performed using SPSS statistical software (version 22.0, Chicago, IL, USA); *p*-values lower than 0.05 were considered statistically significant.

### 2.6. Ethical Considerations

The study was approved by the local ethics committee of “Azienda Ospedaliero Universitaria di Sassari” (Prot No. 1892/13).

## 3. Results

A total of 8894 patients (mean age 52.1 ± 17.3 years; 64.7% female) participated in the study. Among them, 8.5% were found to have a diagnosis of TDs. More specifically, there were 767 patients with AITD (687 with Hashimoto’s thyroiditis and 80 with Graves’ disease) and 451 non-AITD patients. Table 1 shows the features of the study participants.

Patients with TDs were older and overweight compared with controls, but there were no statistically significant differences in terms of smoking habits. The frequency of patients with G6PD deficiency was higher among those with TDs compared with controls, i.e., a value not dissimilar from that previously reported in Northern Sardinia [27].

A separate sub-analysis showed that in both sexes G6PD deficiency was more frequent in patients with AITD than in controls, although it was statistical significant only in females (Table 2).

The results of univariable and multivariable logistic regression analysis, using the presence or absence of AITD as the outcome, according to exposure to G6PD deficiency, among other variables, are shown in Table 3.

The female sex was a strong risk factor and BMI was a borderline risk factor. G6PD deficiency resulted in a significant risk factor both in the univariable analysis and after adjusting for all covariates. The ORs remained statistically significant after stratification by sex in males and females with G6PD deficiency compared with patients with normal enzyme activity (Table 4).

In addition, the association with G6PD deficiency was independently tested in Hashimoto’s thyroiditis and Graves’ disease; a trend for increased risk was similarly found in carriers of the enzyme defect in Hashimoto’s thyroiditis (OR, 1.62; 95% CI 0.92–2.86). However, the small number of patients with Graves’ disease (*n* = 80) did not allow for reliable results.

Finally, the logistic regression model was applied to non-AITD patients. After adjusting for the covariates, G6PD deficiency and non-AITD were not found to be significantly associated (OR, 1.24; 95% CI 0.97–1.58).

## 4. Discussion

This study is the first systematic investigation of a significant number of patients. The results obtained seem to indicate that the inherited condition of G6PD deficiency should be regarded as a significant risk factor for AITD, whereas this association was not observed in the non-AITD group. At the moment, we cannot provide a definite causal explanation. However, some hypotheses can be formulated. In most cells, including thyrocytes, the enzyme G6PD oxidizes glucose-6-phosphate producing NADPH to provide reducing equivalents for the antioxidant defense, namely, to maintain a high intracellular GSH/GSSG ratio [1]. Thyrocytes possess an efficient antioxidant system, able to modulate the continuous production of hydrogen peroxide, which participates in the synthesis of thyroid hormones. Consequently, a tentative hypothesis to explain our findings could be that G6PD deficiency, via intracellular depletion of NADPH, may hamper the conversion of GSSG to GSH necessary to dispose massive intracellular H_2_O_2_ production. In thyrocytes, as in other cells, the tripeptide glutathione (γ-L-glutamyl-L-cysteinylglycine) is synthesized by the enzyme glutathione synthetase (EC 6.3.2.3), but is rapidly oxidized into disulfide (GSSG) along with the inactivation of H_2_O_2_ in the reaction catalyzed by glutathione peroxidase (EC 1.11.1.9) [28]. Hence, GSH must be continuously regenerated by glutathione reductase (EC 1.8.1.7), a NADPH-dependent enzyme [29]. When the availability of NADPH is low (e.g., because of a structurally inefficient G6PD), most of the glutathione remains in the oxidized (disulfide) form and cannot dispose hydrogen peroxide, or serve as cofactor for deiodinases, the enzymes responsible for the transformation of thyroxine (T4) into triiodothyronine (T3).

G6PD deficiency might also enhance AITD risk via other mechanisms, such as a dysregulation of the inflammatory response. Numerous in vitro studies have demonstrated that G6PD-deficient cells release an excess of regulatory cytokines, such as transforming growth factor beta (TGF-β) [30,31]. In general, TGF-β plays a key role in inflammation and oxidative stress, as confirmed by the lowering ROS effect of TGF-β inhibitors [31]. Recent evidence suggests that upregulation of this multifunctional cytokine is crucial in the development of AITD [32]. Through its action on pivotal mechanisms of innate (natural killer cells) and adaptive (Tregs cells) immunity, TGF-β upregulation may contribute to the autoimmune response mounted in AITD. However, TGF-β exerts both pro- and anti-inflammatory effects, and its role in AITD could be more complex than one might think. This intriguing hypothesis, supported only by in vitro evidence, requires confirmation through in vivo studies and, above all, through studies conducted in humans. This hypothesis is consistent with the findings of our study, which indicate an association between G6PD deficiency and AITD, but not with thyroid diseases of different etiology.

The situation can be exacerbated by conditions causing GSH exhaustion in thyrocytes. The most dangerous among these conditions is selenium insufficiency. Selenium is a trace element necessary for the glutathione peroxidase and thioredoxin systems to function in antioxidant defense. Selenium concentration is higher in the thyroid gland than elsewhere in the body tissues, but selenium insufficiency may frequently occur in areas where this element is poorly available in the organic soil [33], including Northern Sardinia [34].

Dietary selenium deficiency may aggravate the oxidative overload in the thyroid gland in G6PD-deficient individuals, thereby increasing the risk of developing AITD [35]. Indeed, this could have been a peculiar condition in our cohort from Northern Sardinia, where a latent selenium deficiency may have acted as an additional risk for thyroid function.

Additional possible risks might include infections (including those from SARS-CoV-2 [36]), drugs, such as paracetamol [37], intake of sweeteners, such as aspartame [38], organic pollutants [39], endocrine disruptors [40] and diabetes [41], to mention a few.

In opposition to this hypothesis, it could be argued that NADPH depletion in the thyroid follicle would also lead to a lower production of H_2_O_2_ via DUOX2 activity. Moreover, NADPH is synthesized in the mitochondrial matrix through alternative biochemical reactions catalyzed by isocitrate dehydrogenase 1, glutamate dehydrogenase, malic enzyme and nicotinamide nucleotide transhydrogenase [42]. However, it could be observed that the production of H_2_O_2_ is so increased in the thyroid follicles due to the actions of enzymes such as NOX4, that the NADPH depletion induced by G6PD deficiency could not be sufficient to significantly inhibit the flow of H_2_O_2_, but enough to hinder glutathione-dependent antioxidant activity. However, these hypotheses must be substantiated using a more solid experimental basis.

This study is only preliminary, and has some limitations due to its retrospective design and the lack of molecular confirmation of G6PD deficiency. Yet, the frequency of G6PD deficiency, as well as of TDs among screened subjects, was comparable to that reported for the general Sardinian population [43], making an association bias unlikely.

## 5. Conclusions

Thyroid diseases are very common, especially in females. They can have a significant impact on metabolism and emotional health, substantially modifying patients’ quality of life. However, they are also pharmacologically curable conditions. The identification of G6PD deficiency as a risk factor provides the opportunity for physicians to make early diagnoses based on G6PD status and to establish appropriate therapy.

## Figures and Tables

**Table 1:** Features of the 8894 study participants with and without TDs.

Variables	Patients with TDs ^§^ (*n* = 1218)	Patients without TDs (*n* = 7676)	*p*-Value
Sex, *n* (%)			
Male Female	131 (10.8) 1087 (89.2)	3035 (39.5) 4641 (60.5)	<0.0001
Mean age, years	55.1 ± 14.9	52.6 ± 17.4	0.001
Smoking habits, *n* (%)			
No	685 (56.2)	4223 (55.0)	0.438
Current or former smoker	533 (43.8)	3453 (45.0)	
Body mass index, kg/m^2^			
<25	678 (55.7)	4568 (59.5)	
25–29.5	392 (32.2)	2266 (29.5)	0.040
≥30	148 (12.2)	842 (11.0)	
G6PD deficiency, *n* (%)			
None	1014 (83.3)	6818 (88.8)	
Yes	204 (16.7)	858 (11.2)	<0.0001

^§^ Thyroid disorders

**Table 2:** Frequency of G6PD deficiency among study participants with autoimmune thyroid diseases (AITD) and controls stratified by sex.

Variables	Males		Females	
	AITD (*n* = 72)	Controls (*n* = 3035)	AITD (*n* = 695)	Controls (*n* = 4641)
G6PD deficiency, *n* (%)				
None	60 (83.3)	2780 (91.6)	577 (83.0)	4057 (87.4)
Yes	12 (16.7) *	255 (8.4)	118 (17.0) *	584 (12.6)

* *p* < 0.05.

**Table 3:** Unadjusted and adjusted Odds Ratios for autoimmune thyroid disease (AITD) among patients included in the study.

Variables	AITD (*n* = 767)	Controls (*n* = 7676)	Unadjusted OR (95% CI ^#^)	Adjusted OR (95% CI ^#^)
Sex, *n* (%) Male Female				
	72 (9.4)	3035 (39.5)	1.00	1.00
	695 (90.6)	4641 (60.5)	6.29 (4.91–8.06) **	6.34 (4.95–8.13) **
Age, *n* (%)				
<60 years ≥60 years	484 (63.1)	4719 (61.4)	1.00	1.00
	283 (36.9)	2957 (38.5)	0.92 (0.79–1.07)	0.97 (0.83–1.14)
Smoking habits, *n* (%) No Current/former smoker				
	429 (55.9)	4223 (55.0)	1.00	1.00
	338 (44.1)	3453 (45.0)	0.98 (0.84–1.13)	1.06 (0.91–1.23)
Body mass index, kg/m^2^				
<25	441 (57.5)	4568 (59.5)	1.00	1.00
25–29.5	237 (30.9)	2266 (29.5)	1.09 (0.93–1.29)	1.26 (1.06–1.49) *
≥30	89 (11.6)	842 (11.0)	1.10 (0.86–1.39)	1.17 (0.91–1.50)
G6PD deficiency, *n* (%)				
None	637 (83.1)	6818 (88.8)	1.00	1.00
Yeas	130 (16.9)	858 (11.2)	1.62 (1.32–1.98) **	1.43 (1.17–1.76) *

^#^ Confidence interval, * *p* < 0.05; ** *p* < 0.001.

**Table 4:** Unadjusted and adjusted odds ratio (OR) for autoimmune thyroid disease (AITD) among 8696 patients stratified by sex.

Variables	Males		Females	
	Unadjusted OR (95% CI ^#^)	Adjusted OR (95% CI ^#^)	Unadjusted OR (95% CI ^#^)	Adjusted OR (95% CI ^#^)
Age, *n* (%)				
<60 years ≥60 years	1.00	1.00	1.00	1.00
	1.16 (0.73–1.86)	1.15 (0.72–1.85)	0.98 (0.84–1.16)	0.96 (0.82–1.14)
Smoking habits, *n* (%) No Current/former smoker				
	1.00	1.00	1.00	1.00
	0.88 (0.55–1.40)	0.91 (0.56–1.47)	1.11 (0.94–1.30)	1.07 (0.91–1.26)
Body mass index, kg/m^2^				
<25	1.00	1.00	1.00	1.00
25–29.5	0.93 (0.56–1.54)	0.94 (0.56–1.57)	1.30 (1.05–1.55) *	1.29 (1.08–1.55) *
≥30	0.67 (0.28–1.58)	0.67 (0.28–1.62)	1.23 (0.96–1.61)	1.23 (0.95–1.59)
G6PD deficiency, *n* (%)				
None	1.00	1.00	1.00	1.00
Yes	2.20 (1.17–4.15) *	2.18 (1.16–4.12) *	1.35 (1.09–1.68) **	1.35 (1.09–1.68) **

^#^ Confidence interval, * *p* < 0.05; ** *p* < 0.001.

## Data Availability

Data will be made available upon specific request to the corresponding author.
